# ROGUE: an R Shiny app for RNA sequencing analysis and biomarker discovery

**DOI:** 10.1186/s12859-023-05420-y

**Published:** 2023-07-29

**Authors:** Alvin Farrel, Peng Li, Sharon Veenbergen, Khushbu Patel, John M. Maris, Warren J. Leonard

**Affiliations:** 1 Laboratory of Molecular Immunology, National Heart, Lung, and Blood Institute, National Institutes of Health, Bethesda, MD USA; 2 Immunology Center, National Heart, Lung, and Blood Institute, National Institutes of Health, Bethesda, MD USA; 3 Division of Oncology and Center for Childhood Cancer Research, Children’s Hospital of Philadelphia, Philadelphia, PA USA; 4 Department of Biomedical and Health Informatics, Children’s Hospital of Philadelphia, Philadelphia, PA USA; 5 Laboratory of Pediatric Gastroenterology, Erasmus University Medical Center, Rotterdam, The Netherlands; 6 Perelman School of Medicine at the University of Pennsylvania, Philadelphia, PA USA; 7 Present Address: Laboratory of Medical Immunology, Department of Immunology, Erasmus University Medical Center, Rotterdam, The Netherlands

**Keywords:** R Shiny, RNA-Seq, Differential expression, Biomarkers, GSEA, Gene ontology

## Abstract

**Background:**

The growing power and ever decreasing cost of RNA sequencing (RNA-Seq) technologies have resulted in an explosion of RNA-Seq data production. Comparing gene expression values within RNA-Seq datasets is relatively easy for many interdisciplinary biomedical researchers; however, user-friendly software applications increase the ability of biologists to efficiently explore available datasets.

**Results:**

Here, we describe ROGUE (RNA-Seq Ontology Graphic User Environment, https://marisshiny.research.chop.edu/ROGUE/), a user-friendly R Shiny application that allows a biologist to perform differentially expressed gene analysis, gene ontology and pathway enrichment analysis, potential biomarker identification, and advanced statistical analyses. We use ROGUE to identify potential biomarkers and show unique enriched pathways between various immune cells.

**Conclusions:**

User-friendly tools for the analysis of next generation sequencing data, such as ROGUE, will allow biologists to efficiently explore their datasets, discover expression patterns, and advance their research by allowing them to develop and test hypotheses.

**Supplementary Information:**

The online version contains supplementary material available at 10.1186/s12859-023-05420-y.

## Background

RNA sequencing (RNA-Seq) has become an extremely powerful tool for understanding biological pathways and molecular mechanisms. Technological advancements, both wet-lab and computational, have transformed RNA-Seq into a more accessible tool, giving biomedical researchers access to a less biased view of RNA biology and transcriptomics [1–3]. The growing power and ever decreasing cost of RNA-Seq technologies have resulted in a marked increase in RNA-Seq dataset production.

The explosion of computational algorithms and pipelines in the last decade has given researchers the ability to perform rigorous analyses and explore RNA-Seq data [4–9]. Differential expression analysis (DEA) [10–13], which is the most common analysis performed on RNA-Seq, is used to estimate steady-state mRNA levels. There are multiple bioinformatics pipelines and packages used to perform DEA [13], including edgeR [10], DESeq[11], and limma-voom [12]. Different combinations of the various algorithms to analyze sequence reads and perform DEA can affect the biological conclusions drawn from the data [7, 14–16]. Researchers must carefully select the optimal combination of tools based on their specific biological questions and the available computational resources to perform deep dives and thorough exploration of their RNA-Seq data [7].

DEA is often combined with gene ontology (GO) analysis, pathway analysis, and clustering algorithms to characterize data and elucidate the processes and dynamics involved in transcription [17]. These studies give new insights into gene regulatory networks and expression. Gene enrichment analysis is a standard GO approach to evaluate upregulated pathways and processes [17–20]. Dimensionality reduction methods, such as multidimensional scaling (MDS) [10, 21], principal component analysis (PCA) [22, 23], and t-distributed stochastic neighbor embedding (t-SNE) [24], are used to identify RNA-Seq libraries with similar gene expression profiles. Moreover, while many other sophisticated RNA-Seq technologies exist, such as isoform analyses, single-cell RNA-Seq, and spatially resolved RNA-Seq methods, bulk RNA-Seq remains a powerful tool that continues to shape our understanding of biology.

The availability of RNA sequencing datasets is becoming more common due to increased support of open data by academicians and requirements by scientific journals and funding agencies to make publication-affiliated datasets publicly available. This has gifted the scientific community with an extensive repository of datasets [25–27] derived from cell lines, animal models, and patient-derived samples of a wide variety of tissues and diseases. Researchers can explore these datasets of interest to generate or test hypotheses. However, even standard DEA and GO analyses often requires a bioinformatician or a computationally savvy biologist.

User-friendly tools for RNA-Seq analyses will allow biomedical scientists with limited programming experience to explore these datasets. Here we present RNA-Seq Ontology Graphic User Environment (ROGUE), an R Shiny application that allows biologists to perform differentially expressed gene analysis, gene ontology and pathway enrichment analysis, potential biomarker identification, and advanced statistical analyses. We demonstrate the capability of ROGUE by exploring the basic differences between CD4^+^ T cells, CD8^+^ T cells, and natural killer (NK) cells. Furthermore, we show how ROGUE can be used to identify biomarkers and differentially enriched pathways present in similar immune cells in different diseases.

We propose that ROGUE will allow scientists to explore their datasets and also compare their findings with publicly available datasets, increasing the potential of data-driven biomedical discovery.

## Methods

### Workflow

**Fig. 1:**
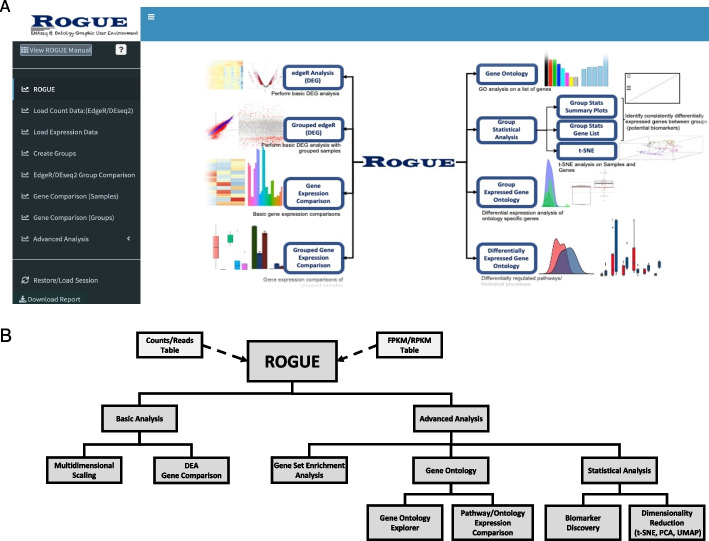
**A** ROGUE R Shiny app graphic user interface. **B** ROGUE workflow. ROGUE takes raw read counts, normalized counts, or quantified expression values (RPKM, FPKM, TPM) as input. The user can quickly look at the clustering of all samples based on the expression values of all genes, perform differential expression analysis, and compare genes between samples or groups. ROGUE also includes statistical tools for gene set enrichment analysis (GSEA), gene ontology (GO) analysis, biomarker discovery, and dimensionality reduction by t-SNE, PCA, or UMAP

ROGUE is an R Shiny web app with a graphic user interface (GUI) (Fig. 1A) that takes expression data as input such as raw read counts, length-normalized counts, expression units including fragments per kilobase of transcript per million mapped reads (FPKM), reads per kilobase of transcript per million mapped reads (RPKM), and transcripts per million (TPM). Users can generate their own RNA-Seq matrix or download publicly available RNA-Seq expression data from databases such as gene expression omnibus (GEO) [25], ArrayExpress [26], The genotype tissue expression (GTEx) Project [27], and the cancer genome atlas (TCGA) [28]. An online manual is available at https://marisshiny.research.chop.edu/ROGUE/Instructions.pdf. When the input is raw read counts or length-normalized counts quantified by packages such as HT-seq [29] or RSEM [8], ROGUE generates RPKM tables and can perform DEA using edgeR [10] or DESeq2 [11] which are two of the state-of-the-art R packages for DEA analysis [13] and has been shown to outperform other methods in various applications [30, 31]. ROGUE also allows users to perform more advanced analyses such as biomarker discovery based on gene expression, dimensionality reduction, gene set enrichment analysis, and gene ontology analysis (Fig. 1B).

Gene expression comparison between samples and groups can be visualized with heatmaps, bar plots, and boxplots. Users can also use ROGUE to predict possible biomarkers by ranking genes with maximized fold change and minimized coefficients of variation in gene expression between groups of samples. The Welch’s t-test and the Wilcoxon Rank Sum Test can also be used to rank genes by their difference in expression distribution between the groups using the Biomarker Discovery Tool.

Gene set enrichment analysis (GSEA) is a computational method that determines whether a pre-ranked (i.e., log fold change) gene list shows statistically significant, concordant differences between two biological states (e.g., CD4^+^ vs. CD8^+^ T cells). GSEA between individual samples or groups can be performed using the Fast Gene Set Enrichment Analysis (fgsea) R package [32] with data imported from the Molecular Signatures Database (MSigDB) [18, 33]. Alternatively, gene ontology analysis on a list of differentially expressed genes can be performed using the Gene Ontology Resource [17, 34], which is imported into ROGUE. Furthermore, ROGUE can determine differentially expressed gene sets using the Gene Ontology Resource. This resource uses the Wilcoxon rank sum test to determine if the expression of all genes within a biological process or molecular function are statistically different between samples or groups.

Dimensionality reduction methods can be applied to the datasets and visualized using 2-dimensional and 3-dimensional plots. ROGUE performs PCA using the ‘prcomp’ R function, t-SNE using the ‘Rtsne’ R package [35], and Uniform Manifold Approximation and Projection (UMAP) method for dimensionality reduction using the ‘uwot’ R package [36–38].

The source code for ROGUE is available at https://github.com/afarrel/ROGUE. All packages and implementation of the tools are described at this repository.

### Datasets

We performed basic analyses on datasets GSE60424 [39], GSE102317 [40], and GSE40350 [41] and GSE101470 [42] from the GEO Database to illustrate the basic features of ROGUE. Human CD4^+^ and CD8^+^ T cells, NK cells, neutrophils, and monocytes from healthy subjects and subjects diagnosed with type 1 diabetes, amyotrophic lateral sclerosis, sepsis, and multiple sclerosis were retrieved from GSE60424. RNA-Seq data from mouse CD4^+^ and CD8^+^ T cells and NK cells were retrieved from GSE102317, GSE40350, and GSE101470, respectively, for additional analyses. Dataset GSE102317 contains RNA-Seq data from CD4^+^ T cells treated with IL-2 and IL-21 for 0 (control), 2, 4, and 24 h. Dataset GSE40350 contains CD8^+^ T cells treated with IL-2 and IL-15 for 0 (control), 4, and 24 h. Dataset GSE101470 includes RNA-Seq from mature CD11b^−^/CD27^−^, CD11b^−^/CD27^+^, CD11b^+^/CD27^+^, and CD11b^+^/CD27^low^ NK cells as well as *Stat5* double knock-in mice with N-terminal mutations in STAT5A and STAT5B that prevent STAT5 tetramerization but not dimerization.

## Results and discussion

### Workflow

We demonstrate the capability of ROGUE by exploring some basic differences between CD4^+^ T cells, CD8^+^ T cells, and natural killer (NK) cells in datasets downloaded from the GEO Database. First, we performed DEA using edgeR [10] and compared the expression of genes of interest between cell types. We then performed GSEA, GO analysis, and biomarker discovery based on gene expression to understand functional differences between the cells and discover possible biomarkers. We used ROGUE to perform dimensionality reduction by t-SNE to evaluate if the transcriptome of these cells were distinct enough to cluster each sample by cell type. Finally, we searched for differentially expressed gene sets from the GO Resource to evaluate changes in pathways pre and post-interferon beta (IFNβ) treatment in immune cells from patients with multiple sclerosis (MS).

### Basic DEA and GO analysis

**Fig. 2:**
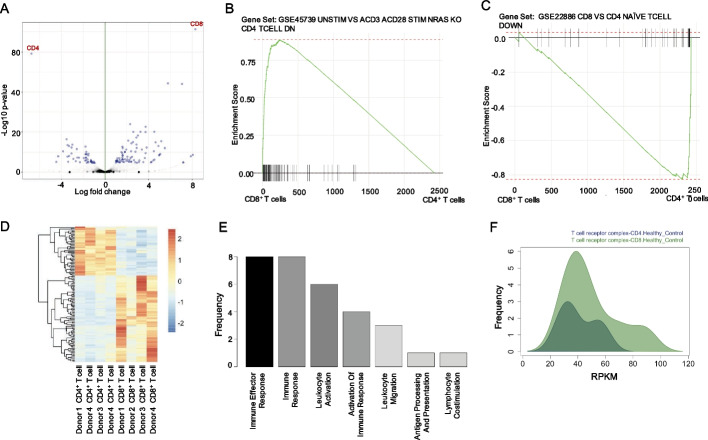
Basic analysis of CD4^+^ T cells versus CD8^+^ T cells in healthy individuals. **A** Volcano plot showing differentially expressed genes. **B** GSEA showing the most enriched gene set when CD8^+^ T cells were compared to CD4^+^ T cells. **C** GSEA shows that the gene set downregulated in naive CD8^+^ T cells when compared to naive CD4^+^ T cells followed the same pattern in the current dataset. **D** Heatmap showing top differentially expressed genes between CD8^+^ and CD4^+^ T cells. **E** Gene ontologies of genes upregulated in CD8^+^ T cells. **F** Distribution of expressed genes related to the T cell receptor complex

To illustrate the basic features of ROGUE, we first performed DEA on CD4^+^ T cells versus CD8^+^ T cells from healthy humans in dataset GSE60424 using edgeR [10] and generated a volcano plot showing the differentially expressed genes (Fig. 2A). We next performed GSEA using the ‘fgsea’ R package to identify enriched gene signatures from the differentially expressed genes between CD4^+^ T cells and CD8^+^ T cells from healthy humans (Fig. 2B, C, Additional file 1). For this illustration, we expect to see gene sets with enhanced expression in experiments with stimulated CD8^+^ T cells or with lower expression in CD4^+^ T cells to be enriched in our CD8^+^ T cells RNA-Seq libraries and lower in our CD4^+^ T cell libraries. Interestingly, the most enriched gene set for CD8^+^ T cells when compared to CD4^+^ T cells was a set (GSE45739) of genes downregulated in CD4^+^ T cells with *Nras* knockout (KO) mice (Fig. 2B). While CD4^+^ thymocyte differentiation is not affected in *Nras* KO mice, CD8^+^ thymocyte differentiation has been shown to be significantly reduced [43]. Not surprisingly, the most enriched gene set for CD4^+^ human T cells, was a set (GSE22886) of genes downregulated in naïve CD8^+^ T cells when compared to CD4^+^ T cells (Fig. 2C). A heatmap was used to display the distinct expression patterns of the differentially expressed genes between CD4^+^ and CD8^+^ T cells from the four healthy donors in the dataset (Fig. 2D). Basic GO analysis of genes upregulated in CD8^+^ T cells showed enrichment in genes related to immune effector process, immune response, and leukocyte activation (Fig. 2E). We next used the gene ontology comparison tool to evaluate which type of T cell expresses more genes related to the T cell receptor (TCR) complex. This analysis interestingly revealed that the TCR complex was more enriched in the CD8^+^ T cells as they expressed more genes at greater RPKM than the CD4^+^ T cells (Fig. 2F).

### Biomarker discovery

**Fig. 3:**
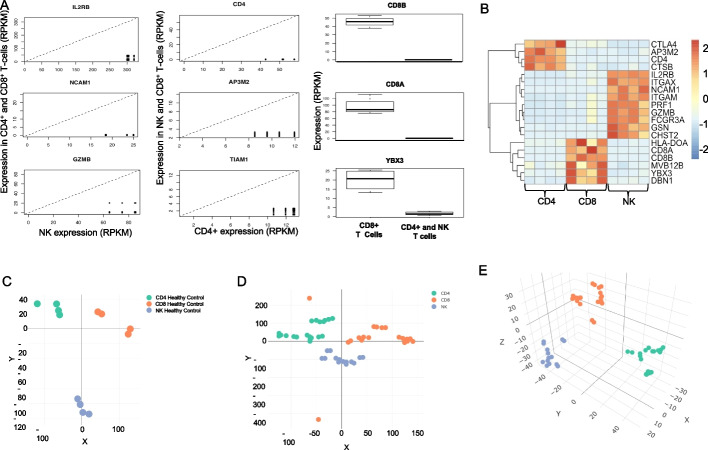
Biomarker Discovery among CD4^+^ T cells, CD8^+^ T cells, and NK cells. **A** The Biomarker tool shows genes with high expression in one cell type and very low expression in the other cell types, suggesting that they may be potential biomarkers. **B** Heatmap showing relative expression values of the potential biomarkers’ gene expression for CD4^+^ T cell, CD8^+^ T cell, and NK cell groups. **C** 2-dimensional t-SNE plot of CD4^+^ T cells, CD8^+^ T cells, and NK cells from healthy controls using the identified potential biomarkers. **D** 2-dimensional t-SNE plot of CD4^+^ T cells, CD8^+^ T cells, and NK cells from both healthy and diseased groups using the identified potential biomarkers. **E** 3-dimensional t-SNE plot using the identified potential biomarkers emphasizes separation between clusters

Biomarker discovery is essential in biomedical and pharmaceutical research [44–46]. Although mRNA is not always translated into protein, one can infer potential biomarkers from RNA-Seq data. ROGUE uses an optional combination of the coefficient of variation (CV), Wilcoxon-ranked sum test, or t-test for biomarker discovery between RNA-Seq library groups. ROGUE was used to identify potential biomarkers between CD4^+^ T cells, CD8^+^ T cells, and NK cells using the Biomarker Discovery tool (Fig. 3A), and a subset of these potential biomarkers was compared across the various cell types using a heatmap (Fig. 3B). The expression values of the potential biomarkers were used to perform t-SNE on all the RNA-Seq libraries. A 2-dimensional plot of the t-SNE results shows that RNA-Seq libraries from CD4^+^ T cells, CD8^+^ T cells, and NK cells from healthy controls clustered reasonably well based on the potential biomarkers discovered (Fig. 3C). Clusters were not as distinct when t-SNE was performed on T and NK cell libraries from both healthy controls and patients in 2 dimensions (Fig. 3D), but the clusters in a 3-dimensional plot generated by t-SNE were more homogeneous (Fig. 3E). We evaluated the occurrence of these biomarkers in mouse immune cells and observed that only a few of the biomarkers can be used across all datasets in both species (Additional file 2: A–B). As expected, CD4 and CTLA4 were identified as potential biomarkers for differentiating CD4^+^ T cells from CD8^+^ T cells and NK cells across both datasets while CD8A and CD8B were identified as potential biomarkers for CD8^+^ T cells. Gene expression of the potential human NK cell biomarkers were enriched in mouse NK cells that expressed CD27 (Additional file 2: C–D). t-SNE was performed on the mouse datasets using the gene expressions of the potential biomarkers. The enrichment of the potential human NK cell biomarkers in mouse CD27^+^ NK cells was reflected in the t-SNE plot as they formed a distinct cluster from the other NK cells (Additional file 2: E). It is worth noting that even though the mouse immune cells cluster well using the biomarkers ascertained from the human immune cells, it is possible that the immune cells cluster well due to a batch effect instead of gene expression signature (Additional file 2: F).

### Comparison of biological pathways after treating multiple sclerosis patients with IFNβ

**Fig. 4:**
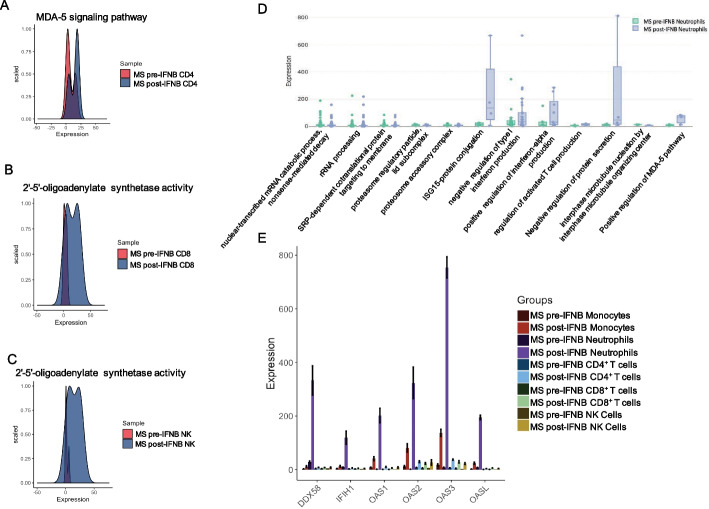
Using differentially expressed pathways to generate and/or explore hypotheses. **A** Distribution of MDA-5 signaling pathway in untreated (red) and IFNβ-treated (blue) CD4^+^ T cells showing an upregulation of genes related to MDA-5 signaling. **B**, **C** Distribution of 2′–5′-oligoadenylate synthetase activity in untreated (red) and IFNβ-treated (blue) CD8^+^ T cells (**B**) and NK cells (**C**) showing an upregulation of genes 2′–5′-oligoadenylate synthetase activity. **D** Boxplots showing pathways that may be differentially regulated in neutrophils with multiple genes consistently upregulated or downregulated post-IFNβ treatment. **E** Bar plot showing upregulated MDA-5 (IFIH1), RIG-I (DDX58), and genes related to 2′–5′-oligoadenylate synthetase in IFNβ-treated monocytes, neutrophils, CD4 + T cells, CD8 + T cells, and NK cells

Dataset GSE60424 contains RNA-Seq data from CD4^+^ T cells, CD8^+^ T cells, NK cells, neutrophils, and monocytes of MS patients before and after IFNβ treatment. MS is an inflammatory demyelinating disease of the central nervous system [47]. IFNβ treatment is a safe and reasonably effective treatment for MS patients [48–51] due to its anti-inflammatory and immunomodulatory effects [52, 53]. While this is a widely-used treatment, the precise mechanism is unknown. To identify potential hypotheses of the mechanism downstream of IFNβ treatment, we used ROGUE to identify differentially expressed biological processes in CD4^+^ T cells, CD8^+^ T cells, and NK cells isolated from patients pre- or post-treatment with IFNβ. CD4^+^ T cells showed upregulation of the MDA-5 signaling pathway, among other biological processes (Fig. 4A and Additional file 3). CD8^+^ T cells and NK cells showed upregulation of 2′–5′-oligoadenylate synthetase activity (Fig. 4B, C). Given that the MDA-5 signaling pathway and 2′–5′-oligoadenylate synthetase activity are both involved in interferon signaling in innate immunity [54–57], and both pathways were upregulated in CD4^+^ T cells, CD8^+^ T cells, and NK cells (Additional file 4: A), we examined the differentially expressed biological processes in neutrophils and monocytes. As expected, we observed an increase in pathways related to interferon production, protein secretion, as well as positive regulation of MDA-5 pathway (Fig. 4D and Additional file 4: B). This led us to examine the expression of genes related to MDA-5 and 2′–5′-oligoadenylate synthetase in all five cell types pre- and post-treatment, as this might give insights into the underlying mechanism. Furthermore, there is at least one report that polymorphisms in MDA-5 (IFIH1) are associated with MS [58], although another report states that this association does not exist in a specific French population [59]. Nevertheless, the MDA-5 signaling pathway and 2′–5′-oligoadenylate synthetase activity were upregulated in all five cell types (Fig. 4E). We then confirmed that both MDA-5 and RIG-I (DDX58) are upregulated in MS patients’ immune cells following treatment with IFNβ, as they are involved in the induction of IRF7 expression and constitutively-expressed IRF3 [57, 60] (Additional file 4: C). A well-defined mechanism of interferon-stimulated gene (ISG) expression is that IRF3 and IRF7 regulate the expression of type 1 interferons, which then induce ISGs through JAK-STAT signaling, including OAS1A and OAS1B [56]. However, IFNα and IFNβ mRNAs are not expressed, which suggests that administered IFNβ rather than endogenously produced IFNβ induces ISGs through the JAK-STAT pathway. This model is consistent with our data, as ISGs were upregulated in all five cell types after IFNβ-treatment with significantly greater expression of MDA-5, RIG-1 and ISGs observed in neutrophils (Fig. 4E and Additional file 4: C). Given that 2′–5′-oligoadenylate synthetase can induce apoptosis in tumors [61], perhaps this alternative role of 2′–5′-oligoadenylate synthetase also occurs in immune cells, giving it a pro-inflammatory role as well as an anti-inflammatory role by promoting apoptosis and regulating cell growth and proliferation [57]. Furthermore, the disproportionate upregulation of genes with pro-apoptotic and antiproliferative roles in neutrophils supports Hasselbalch and Søndergaard’s report of a higher neutrophil-to-lymphocyte ratio, which is a marker of systemic inflammation, before treatment with IFNβ when compared to controls by [62]. Moreover, Pierson et al. demonstrated that depleting neutrophils in the MS animal model reduces the progression of the disease and Naegel et al. showed evidence that the increase in neutrophils in relapsing–remitting MS is likely due to decreased apoptosis [63, 64]. If this potential pro-apoptotic anti-inflammatory role of 2′–5′-oligoadenylate synthetase exists, it could be the mechanism by which IFNβ treatment positively impacts MS patients. In addition to 2′–5′-oligoadenylate synthetase activity, IFNβ may be involved in another pathway that explains the MDA-5/RIG-1 upregulation. Shimoni et al. suggested that IFNβ can bind cell surface receptors and promote the induction of RIG-1 as part of a positive feedback loop [65]. Wang et al. further showed that RIG-1 and MDA5 signaling induces tumor necrosis factor (TNF) in macrophages [66], and TNF has been shown to have anti-inflammatory effects in MS [67]. The anti-inflammatory effects of TNF coupled with the pro-apoptotic role of 2′–5′-oligoadenylate synthetase may be part of the downstream mechanism contributing to the positive response induced by IFNβ in MS patients.

## Conclusion

ROGUE is designed to be a user-friendly R Shiny application that allows users to perform basic tasks with available RNA-Seq data such as differentially expressed gene analysis and gene ontology analysis. While other freely available web tools and portals have been developed to allow researchers to address discrete questions based on molecular and genomic datasets without the need for strong computational skills [68, 69], ROGUE allows deeper dataset exploration, allowing users to compare gene expression and gene set enrichments between samples and groups. For example, users can explore similarities of expression profiles using the dimensionality reduction methods such as t-SNE, PCA, UMAP, and MDS and search for potential biomarkers between groups of RNA-Seq libraries, to our knowledge making it the only currently available tool to allow this range of dataset analysis (Additional file 5). Furthermore, users have the option to download their session and continue their analysis at a later time. Users can also restore a session if the web application gets disconnected from the server. In addition to the case study presented here, we successfully tested ROGUE on ten diverse human and mouse case studies downloaded from Expression Atlas to illustrate the various applications and robustness (Additional file 6). It is worth noting that ROGUE is an R Shiny application thus allowing the inclusion of many statistical and graphical functions by the R community as well as the ability to be implemented on both local and web servers; however, like all R Shiny applications there are limitations. One of these limitations is that R Shiny applications that are implemented on web servers may perform slowly and sometimes disconnect from the server resulting in a subsequent crash when processing large datasets or performing computationally intensive functions. For this reason, we recommend downloading the local version of ROGUE from https://github.com/afarrel/ROGUE when processing large datasets. Here, we show that a user can explore RNA-Seq data obtained from public databases and use ROGUE to analyze that data to generate or support new or existing hypotheses. ROGUE provides non-R programmers access to many statistical and graphical R packages for RNA-Seq analyses through a GUI so they can analyze their data and create figures. Ideally, tools like ROGUE will allow more biomedical researchers to take advantage of genomic data available and help expedite needed bioinformatics analyses. ROGUE is available at https://marisshiny.research.chop.edu/ROGUE/.

## Availability and requirements

Project Name: ROGUE.

Project Home Page: https://marisshiny.research.chop.edu/ROGUE/.

Github: https://github.com/afarrel/ROGUE.

Operating System: Platform independent.

Programming language: R.

Other requirements: R environment and included packages. Tested on R version 3.6.

Any restrictions to use by non-academics: none.

## Supplementary Information

**Additional file 1: **GSEA analysis of healthy human CD8^+^ T cells vs CD4^+^ T cells.

**Additional file 2:** Evaluating biomarkers found in human CD4^+^ T cells, CD8^+^ T cells, and NK cells in mouse immune cells from different datasets.

**Additional file 3:** Distribution of gene expression profiles in the differentially expressed pathways.

**Additional file 4:** Evaluation of MD5A-signaling, RIG-1 signaling, and 2'-5'-oligoadenylate synthetase pre- and post-IFNβ treatment.

**Additional file 5:** Available Rshiny RNAseq analysis tools.

**Additional file 6:** List of case studies.

## Data Availability

RNA sequencing expression data from human immune cells: https://www.ncbi.nlm.nih.gov/geo/query/acc.cgi?acc=GSE60424. RNA sequencing expression data from mouse immune cells: https://www.ncbi.nlm.nih.gov/geo/query/acc.cgi?acc=GSE102317. https://www.ncbi.nlm.nih.gov/geo/query/acc.cgi?acc=GSE40350. https://www.ncbi.nlm.nih.gov/geo/query/acc.cgi?acc=GSE101470

## Abbreviations

CV: Coefficient of variation
DEA: Differential expression analysis
FGSEA: Fast gene set enrichment analysis
FPKM: Fragments per kilobase of transcript per million mapped reads
GEO: Gene expression omnibus
GO: Gene ontology
GSEA: Gene set enrichment analysis
GTEx: The genotype tissue expression
GUI: Graphic user interface
IFNα: Interferon alpha
IFNβ: Interferon beta
ISG: Interferon-stimulated gene
MDS: Multidimensional scaling
MSigDB: Molecular signatures database
MS: Multiple sclerosis
NK: Natural killer
PCA: Principal component analysis
RNA-Seq: RNA sequencing
ROGUE: RNA-Seq ontology graphic user environment
RPKM: Reads per kilobase of transcript per million mapped reads
TCGA: The cancer genome atlas
TCR: T cell receptor
TNF: Tumor necrosis factor
TPM: Transcripts per million
t-SNE: T-distributed stochastic neighbor embedding
UMAP: Uniform manifold approximation and projection
