# Supplementary material for: ROGUE: an R Shiny app for RNA sequencing analysis and biomarker discovery
Source: BMC Bioinformatics. 2023 Jul 29;24:303. doi: 10.1186/s12859-023-05420-y (PMC10386769; doi:10.1186/s12859-023-05420-y)

Additional File 1: GSEA analysis of healthy human $CD8^+$ T cells vs $CD4^+$ T cells. A: Top and bottom 5 enriched gene sets from immunologic gene signatures between healthy human $CD8^+$ and $CD4^+$ T cells (control). B: Top and bottom 5 enriched gene sets from gene ontology gene signatures between healthy human $CD8^+$ and $CD4^+$ T cells (control).

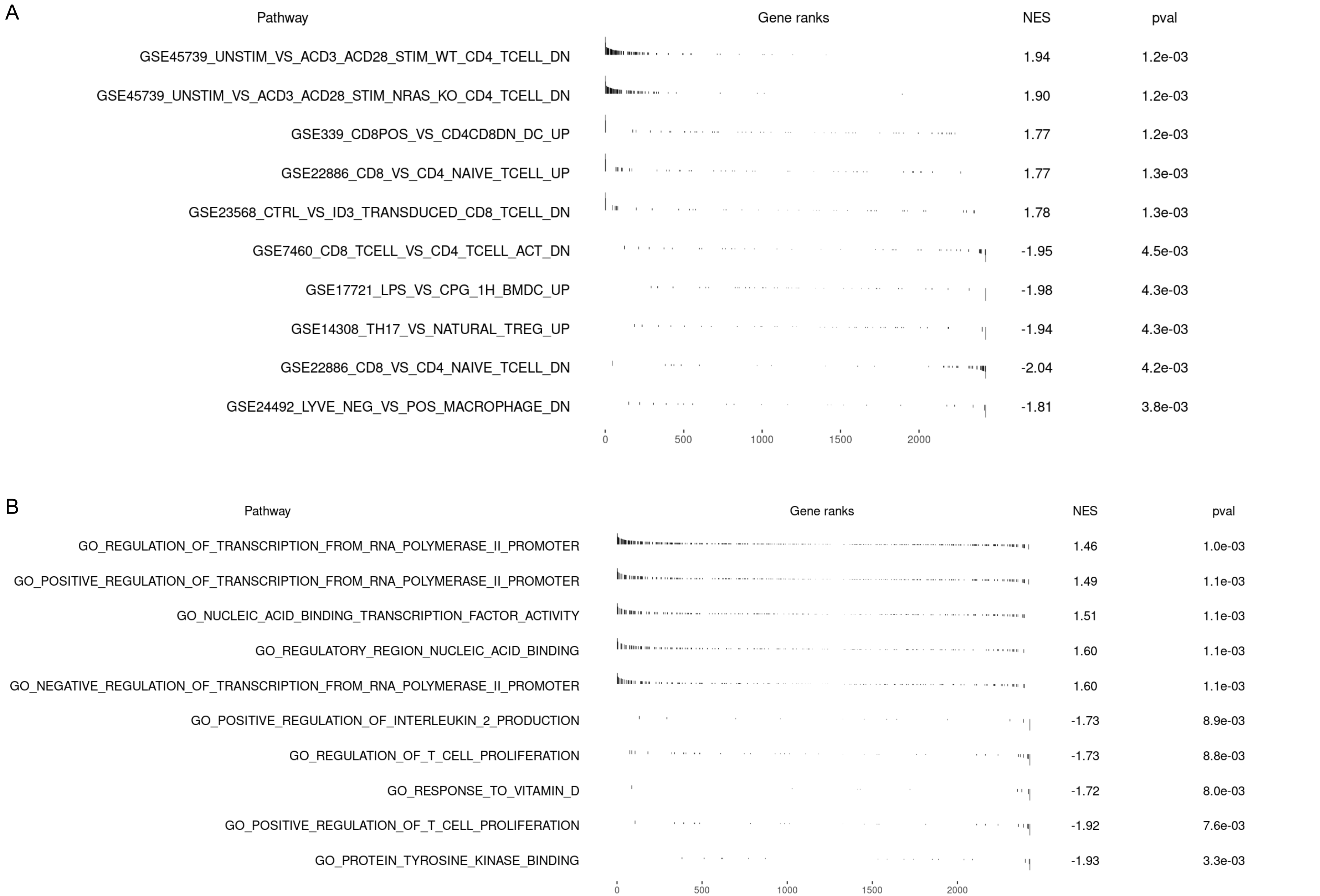

Supplement: Supplementary file 1 — Additional file 1: GSEA analysis of healthy human CD8+ T cells vs CD4+ T cells. [file 12859_2023_5420_MOESM1_ESM.pdf]
