# Supplementary figures and images for: ROGUE: an R Shiny app for RNA sequencing analysis and biomarker discovery
Source: BMC Bioinformatics. 2023 Jul 29;24:303. doi: 10.1186/s12859-023-05420-y (PMC10386769; doi:10.1186/s12859-023-05420-y)

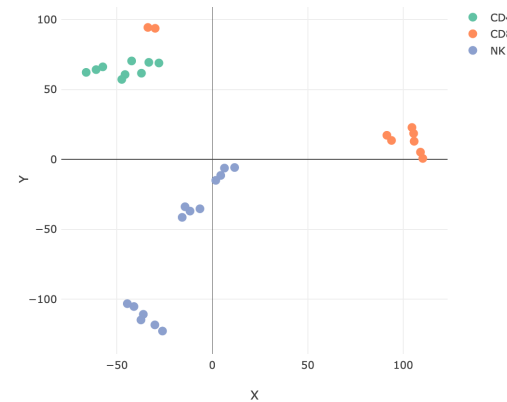

Supplement: Supplementary file 2 — Additional file 2: Evaluating biomarkers found in human CD4+ T cells, CD8+ T cells, and NK cells in mouse immune cells from different datasets. [file 12859_2023_5420_MOESM2_ESM.pdf]
