# Supplementary material for: ROGUE: an R Shiny app for RNA sequencing analysis and biomarker discovery
Source: BMC Bioinformatics. 2023 Jul 29;24:303. doi: 10.1186/s12859-023-05420-y (PMC10386769; doi:10.1186/s12859-023-05420-y)

Additional File 3: Distribution of gene expression profiles in the differentially expressed pathways, as determined by ROGUE using lenient thresholds (log2 Fold Change = 0.4 and Wilcoxon Rank sum test p-value = 0.2) in $CD4^+$ T cells (A), $CD8^+$ T cells (B), and NK cells (C).

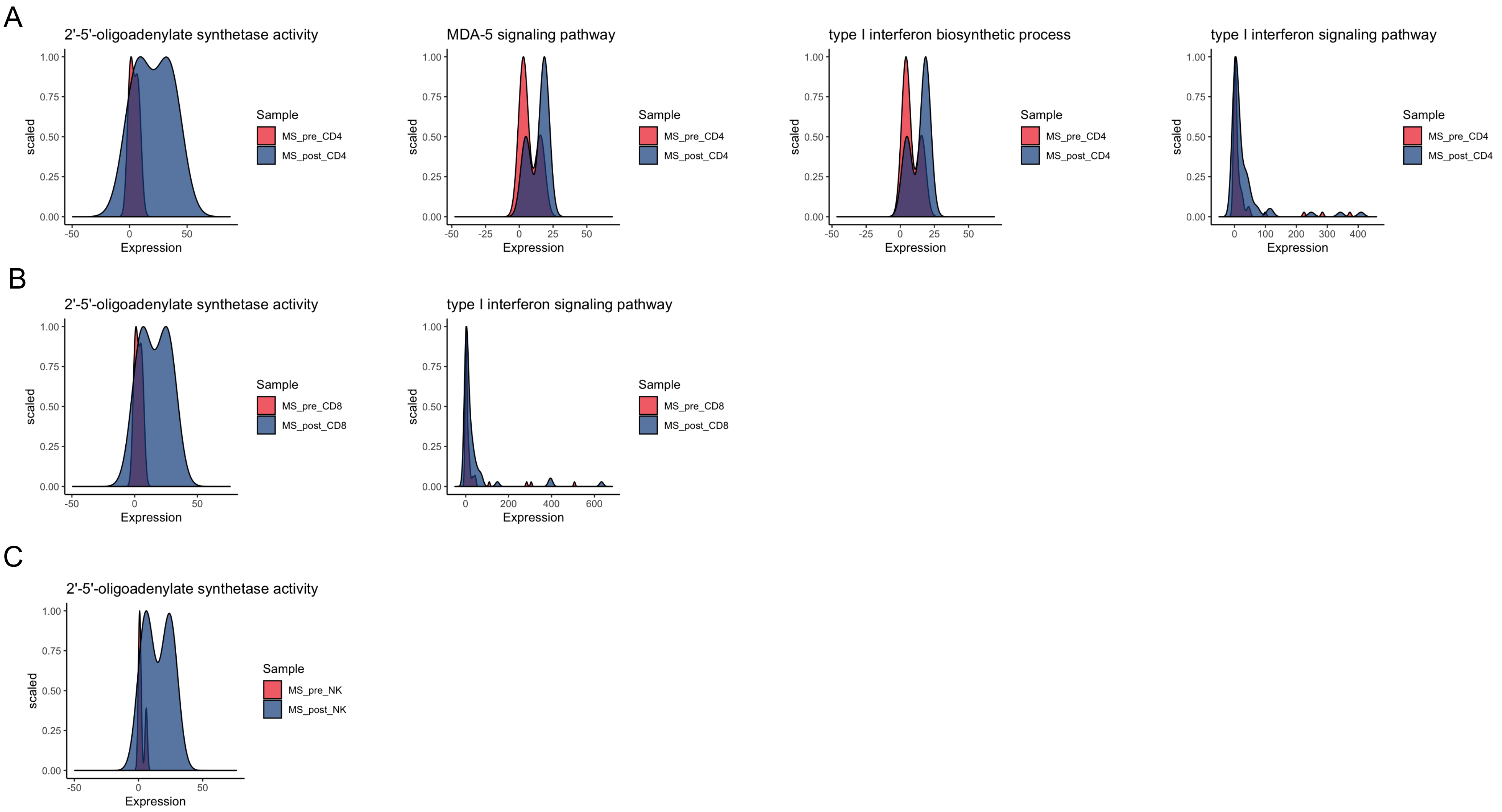

Supplement: Supplementary file 3 — Additional file 3: Distribution of gene expression profiles in the differentially expressed pathways. [file 12859_2023_5420_MOESM3_ESM.pdf]
