# Supplementary material for: ROGUE: an R Shiny app for RNA sequencing analysis and biomarker discovery
Source: BMC Bioinformatics. 2023 Jul 29;24:303. doi: 10.1186/s12859-023-05420-y (PMC10386769; doi:10.1186/s12859-023-05420-y)

Additional File 4: Evaluation of MD5A-signaling, RIG-1 signaling, and 2'-5'-oligoadenylate synthetase pre and post-IFNβ treatment. A: Boxplots showing up regulation of MDA-5 signaling pathway and 2'-5'-oligoadenylate synthetase activity in IFNβ-treated $CD4^{+}$ T cells (red), $CD8^{+}$ T cells (blue), and NK cells (red). B: Boxplots showing pathways that may be differentially regulated in monocytes with multiple genes consistently up or downregulated post-IFNβ treatment. C: Bar plot of expressions of a subset of genes related to MD5A-signaling, RIG-1 signaling, and 2'-5'-oligoadenylate synthetase activity.

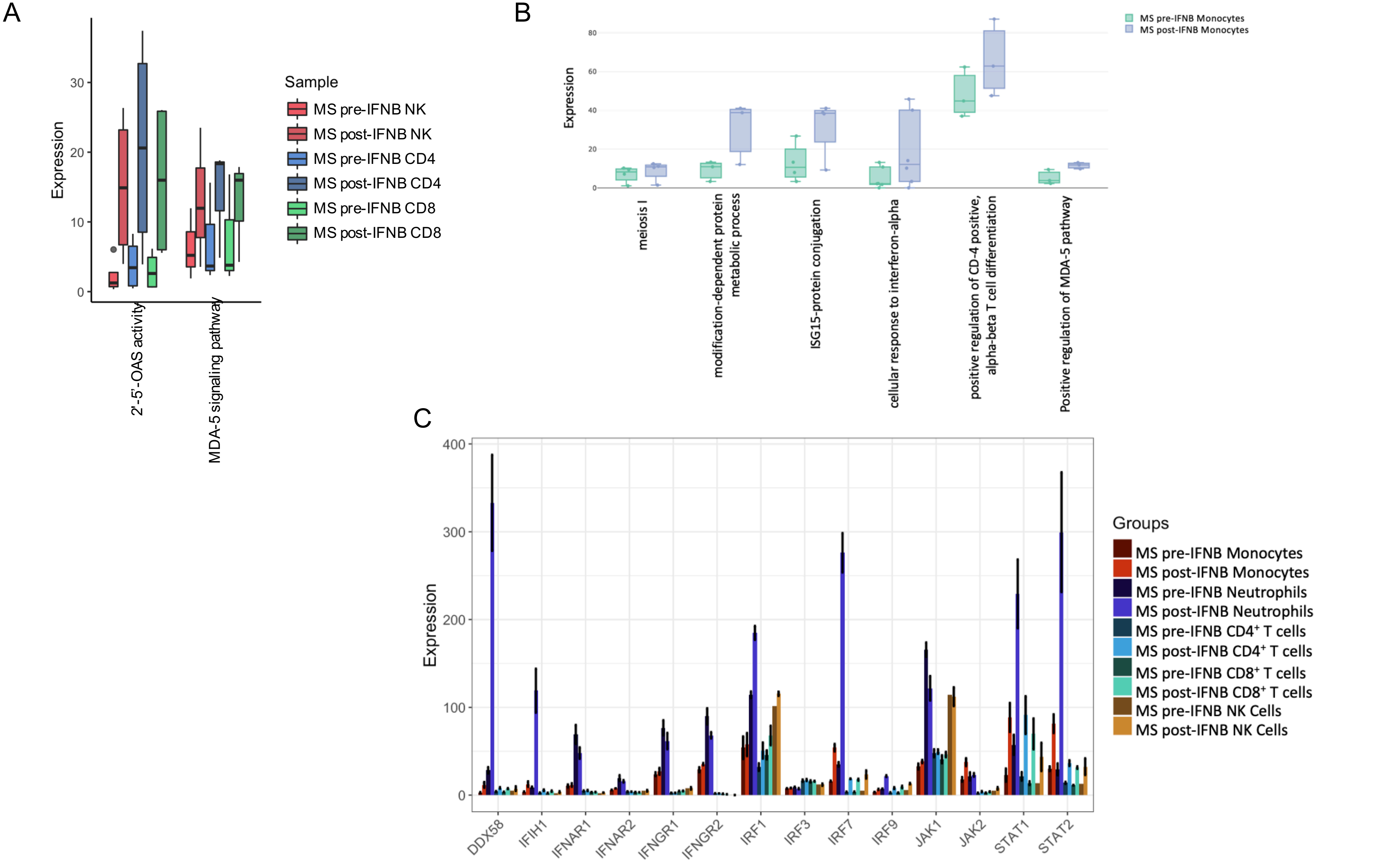

Supplement: Supplementary file 4 — Additional file 4: Evaluation of MD5A-signaling, RIG-1 signaling, and 2'-5'-oligoadenylate synthetase pre- and post-IFNβ treatment. [file 12859_2023_5420_MOESM4_ESM.pdf]
