# Supplementary material for: ROGUE: an R Shiny app for RNA sequencing analysis and biomarker discovery
Source: BMC Bioinformatics. 2023 Jul 29;24:303. doi: 10.1186/s12859-023-05420-y (PMC10386769; doi:10.1186/s12859-023-05420-y)
Supplement: Supplementary file 6 — Additional file 6: List of case studies. [file 12859_2023_5420_MOESM6_ESM.docx]

**Additional File 6 – List of case studies**

Besides the case study presented in the manuscript, we also tested ROGUE on diverse RNA-Seq datasets downloaded from Expression Atlas (<http://www.ebi.ac.uk/gxa>). ROGUE was used successfully to analyze data from 10 case studies (5 Homo sapiens and 5 Mus musculus datasets).

Case study 1: Homo sapiens

[Tissue-dependent transcriptional and bacterial associations in primary sclerosing cholangitis-associated inflammatory bowel disease](https://www.ebi.ac.uk/gxa/experiments/E-MTAB-9658/Results)

Assays: [164](https://www.ebi.ac.uk/gxa/experiments/E-MTAB-9658/Experiment%20Design)

- [disease](https://www.ebi.ac.uk/gxa/experiments/E-MTAB-9658/Experiment%20Design)
- [organism part](https://www.ebi.ac.uk/gxa/experiments/E-MTAB-9658/Experiment%20Design)

Case study 2: Homo sapiens

[BT549 cells depleted of ELP3 compared to control](https://www.ebi.ac.uk/gxa/experiments/E-MTAB-9206/Results)

Assays: [6](https://www.ebi.ac.uk/gxa/experiments/E-MTAB-9206/Experiment%20Design)

- [RNA interference](https://www.ebi.ac.uk/gxa/experiments/E-MTAB-9206/Experiment%20Design)

Case study 3: Homo sapiens

[The effect of very-high-molecular-mass hyaluronan (vHMM-HA) on CD44-overexpressing IMR90 cells](https://www.ebi.ac.uk/gxa/experiments/E-MTAB-8943/Results)

Assays: [9](https://www.ebi.ac.uk/gxa/experiments/E-MTAB-8943/Experiment%20Design)

- [compound](https://www.ebi.ac.uk/gxa/experiments/E-MTAB-8943/Experiment%20Design)
- [genotype](https://www.ebi.ac.uk/gxa/experiments/E-MTAB-8943/Experiment%20Design)

Case study 4: Homo sapiens

[RNA-seq of SH-SY5Y NB cells stably transduced with shETV5 compared to parental cells, cell line samples and samples of xenografted tumors](https://www.ebi.ac.uk/gxa/experiments/E-MTAB-6713/Results)

Assays: [180](https://www.ebi.ac.uk/gxa/experiments/E-MTAB-6713/Experiment%20Design)

- [RNA interference](https://www.ebi.ac.uk/gxa/experiments/E-MTAB-6713/Experiment%20Design)
- [growth condition](https://www.ebi.ac.uk/gxa/experiments/E-MTAB-6713/Experiment%20Design)

Case study 5: Homo sapiens

[CD4+ T Cells Gene Expression-Based Biomarkers in Juvenile Idiopathic Arthritis (JIA)](https://www.ebi.ac.uk/gxa/experiments/E-GEOD-83415/Results)

Assays: [21](https://www.ebi.ac.uk/gxa/experiments/E-GEOD-83415/Experiment%20Design)

- [disease staging](https://www.ebi.ac.uk/gxa/experiments/E-GEOD-83415/Experiment%20Design)

Case study 6: Mus musculus

[RNA-seq analysis of crypt samples isolated from the small intestine of Bach2 KO and control mice](https://www.ebi.ac.uk/gxa/experiments/E-MTAB-9865/Results)

Assays: [6](https://www.ebi.ac.uk/gxa/experiments/E-MTAB-9865/Experiment%20Design)

- [genotype](https://www.ebi.ac.uk/gxa/experiments/E-MTAB-9865/Experiment%20Design)

Case study 6: Mus musculus

[RNA-Seq of pancreatic islets from whole body knockout of lncRNA Pax6os1 mice](https://www.ebi.ac.uk/gxa/experiments/E-MTAB-9213/Results)

Assays: [10](https://www.ebi.ac.uk/gxa/experiments/E-MTAB-9213/Experiment%20Design)

- [genotype](https://www.ebi.ac.uk/gxa/experiments/E-MTAB-9213/Experiment%20Design)

Case study 7: Mus musculus

[RNA-seq of intestinal crypts vs intestinal smooth muscle with a specific MMP17 deletion compared to wild-type](https://www.ebi.ac.uk/gxa/experiments/E-MTAB-9180/Results)

Assays: [12](https://www.ebi.ac.uk/gxa/experiments/E-MTAB-9180/Experiment%20Design)

- [genotype](https://www.ebi.ac.uk/gxa/experiments/E-MTAB-9180/Experiment%20Design)
- [organism part](https://www.ebi.ac.uk/gxa/experiments/E-MTAB-9180/Experiment%20Design)

Case study 8: Mus musculus

[RNAseq of mouse embryonic fibroblasts from conditional knock-in mice carrying a phosphorylation-deficient S180A (SA) mutation in exon 5 of the endogenous mouse Trp53 gene locus](https://www.ebi.ac.uk/gxa/experiments/E-MTAB-9171/Results)

Assays: [6](https://www.ebi.ac.uk/gxa/experiments/E-MTAB-9171/Experiment%20Design)

- [compound](https://www.ebi.ac.uk/gxa/experiments/E-MTAB-9171/Experiment%20Design)

Case study 9: Mus musculus

[RNA-Seq of bone marrow derived macophages (BMDMs) and brain tissue isolated from wild type and Irgm1 knockout mice](https://www.ebi.ac.uk/gxa/experiments/E-MTAB-9164/Results)

Assays: [12](https://www.ebi.ac.uk/gxa/experiments/E-MTAB-9164/Experiment%20Design)

- [genotype](https://www.ebi.ac.uk/gxa/experiments/E-MTAB-9164/Experiment%20Design)
- [organism part](https://www.ebi.ac.uk/gxa/experiments/E-MTAB-9164/Experiment%20Design)

Case study 10: Mus musculus

[Transcriptomic analysis of the distal colon of wild type C57BL/6N mice and mice harboring a mutation (S839I) in Gucy2c](https://www.ebi.ac.uk/gxa/experiments/E-MTAB-9148/Results)

Assays: [8](https://www.ebi.ac.uk/gxa/experiments/E-MTAB-9148/Experiment%20Design)

- [genotype](https://www.ebi.ac.uk/gxa/experiments/E-MTAB-9148/Experiment%20Design)
